# Childhood ischaemic stroke in the basal ganglia can lead to fine motor and anxiety disorders: a retrospective analysis and follow-up of 109 cases

**DOI:** 10.1186/s12883-021-02112-z

**Published:** 2021-02-20

**Authors:** Tianyi Li, Jiannan Ma, Siqi Hong, Yuanyuan Luo, Xiujuan Li, Tingsong Li, Li Jiang

**Affiliations:** Department of Neurology, Ministry of Education Key Laboratory of Child Development and Disorders, National Clinical Research Center for Child Health and Disorders, China International Science and Technology Cooperation Base of Child Development and Critical Disorders, Children’s Hospital of Chongqing Medical University, Chongqing, P.R. China

## Abstract

**Background:**

Stroke in children easily causes long-term dysfunction. Whether the prognoses of motor and anxiety disorders are related to the affected stroke area has not been reported.

**Methods:**

One hundred nine cases of children with ischaemic stroke were reviewed and divided into three groups: lenticular nucleus lesions only (lenticular nucleus group), lenticular nucleus and caudate head lesions (caudate head group), and lenticular nucleus and thalamus lesions (thalamus group). Overall prognosis was evaluated by the mRS score. The SCAS-P was used to evaluate anxiety in children aged ≥6 years.

**Results:**

mRS scores were ≤ 2 points (mean: 0.62), no significant difference among groups. 3/21 (14.2%) patients in the caudate head group changed handedness, which is significantly higher than other groups. Patients with lesions in thalamus group had significantly higher SCAS-P scores.

**Conclusions:**

The overall prognosis of children with basal ganglia ischaemic stroke is good. However, hand preference changes and anxiety disorders may develop. Patients in the caudate head groups are more likely to suffer from fine motor disorders and changes in handedness. Patients within the thalamus group are more prone to anxiety than patients in the other groups. Anxiety disorders should be noted in children with basal ganglia stroke.

## Background

Ischaemic stroke is not a common disease in childhood, during which time children are undergoing physical and psychological development. The sequelae of arterial ischaemic stroke are serious; the incidence rate is high, at approximately 80%, and the mortality rate is approximately 5% [1, 2]. The impact is relatively large and persists for a long time, usually causing psychological and economic burdens on the family. The incidence rate of a long-lasting effect is approximately 1.2–8/100000, which is higher in males, blacks and infants [3, 4]. The aetiology of childhood stroke is essentially different from that of adults. There is almost no atherosclerosis in the aetiology of stroke in children [5, 6].

Basal ganglia ischaemic stroke in children is a special type of stroke that occupies an important proportion of childhood stroke. For a long time, basal ganglia ischaemic stroke was not well understood. Recently, an increasing number of studies have suggested that basal ganglia ischaemic stroke can lead to psychological diseases such as anxiety and depression, which seriously affect the development of children’s physical and mental health as well as affect the long-term quality of life of the children [7, 8]. In addition, the basal ganglia area has a precise structure, and different brain regions play special functions. Most previous studies on basal ganglia stroke in children have not analysed the differences among affected regions. The lenticular artery supplies blood to the head of the caudate nucleus, upper segment of the lenticular nucleus and forelimb of the internal capsule. The anterior choroidal artery supplies blood to the lower segment of the lenticular nucleus, caudate nucleus body, caudate nucleus tail, thalamus and posterior limb of the internal capsule [9]. What is the prognosis and anxiety level of children with basal ganglia ischaemic stroke? Are these factors related to different affected regions? These questions have not yet been answered. To better diagnose and manage children with basal ganglia ischaemic stroke, we retrospectively analysed and followed up with children with basal ganglia ischaemic stroke at our centre.

## Method

This study retrospectively reviewed and followed 109 cases of ischaemic stroke in the basal ganglia and/or thalamus in children from 2005 to 2019 at the Southwest China Neurology Center. This study was approved by the ethics committee of Children’s Hospital of Chongqing Medical University, and consent was obtained from the parents of each child. The follow-up time ranged from 1 to 15 years, with an average of 6.5 years. To make a clear diagnosis and determine the scope of the lesions, all cases were completed with brain computed tomography (CT), magnetic resonance imaging (MRI) and magnetic resonance angiography (MRA) to confirm that no other brain regions except the basal ganglia and/or thalamus were involved. According to the different brain regions involved, they were divided into three groups: only the lenticular nucleus (lenticular nucleus group), the lenticular nucleus and caudate head (caudate head group), and the lenticular nucleus and thalamus (thalamus group). General data, clinical symptoms and imaging manifestations of the patients in each group were retrospectively analysed. Limb muscle strength, fine motor function, facial paralysis and convulsion were followed up. The modified Rankin scale score (mRS) was used to evaluate the overall prognosis. The Spence Children’s Anxiety Scale for Parents (SCAS-P) scale was used to evaluate the anxiety of children aged 6 and above. Two paediatric neurologists conducted physical examinations and data processing on the 109 children. All the parents signed informed consent forms.

### Modified Rankin scale score

To evaluate the outcome, disability was scored using the mRS for children based on information from the clinical examination by the neuropaediatricians. A score 0 indicated no symptoms at all. A score of 1 indicated no significant disabilities despite symptoms; the child exhibited behaviour appropriate to his/her age and normal further development. A score of 2 indicated slight disability; the child was unable to carry out all previous activities, but had the same independence as other age- and sex-matched children (no decrease in the gross motor function scale level). A score of 3 denoted moderate disability; the child required some help but was able to walk without assistance. In younger patients, this score also indicated adequate motor development despite mild functional impairment (reduction of 1 level on the gross motor function scale). A score of 4 indicated moderately severe disability; the child was unable to walk without assistance. In younger patients, this also entailed a reduction of at least 2 levels on the gross motor function scale. A score of 5 signified severe disability; the child was bedridden, requiring constant nursing care and attention. A score of 6 was indicated the child was dead.

### Spence Children’s Anxiety Scale for Parents

To determine the degree of anxiety in children with basal ganglia and/or ischaemic stroke, we used the 38-item Chinese version of Spence Children’s Anxiety Scale for Parents [10]. The SCAS-P is a parent-report measure designed to assess anxiety in children older than 6 years [11]. It included six subscales:

Separation anxiety (SAD; items 5, 8, 11, 14, 15, and 38), social phobia (SoP; items 6, 7, 9, 10, 26, and 31), obsessive-compulsive disorder (OCD; items 13, 17, 24, 35, 36, and 37), panic and agoraphobia, (PA; items 4, 19, 25, 27, 28, 30, 32, 33, and 34), fear of physical injuries (PhF; items 12, 16, 21, 23, and 29), and generalized anxiety disorder (GAD; items 1, 2, 3, 18, 20, and 22).

Parents completed the questionnaire, responding truthfully to the items according to their own child’s situation by scoring each item from 0 to 3 (0 = never, 1 = sometimes, 2 = usually, 3 = always). The scores of the total scale was calculated by adding the responses of the relevant items, with higher numbers reflecting greater anxiety. The Chinese version of the SCAS-P has demonstrated adequate internal consistency. In this study, 86 children were more than 6 years old at follow-up, and the Cronbach’s alpha of this study was 0.829.

### Statistics

SPSS 20.0 software was used to analyse the data. Unpaired t test, fisher exact test, chi square test and one-way ANOVA test were used. *P* < 0.05 was considered to indicate a significant difference.

## Results

There were 109 children with basal ganglia and/or thalamus ischaemic stroke, accounting for 14.2% (109/768) of all children with ischaemic stroke in our centre all together. In this group, 64/109 male patients accounted for 58.7%, and 45/109 cases were female, accounting for 41.3% of the cases. The onset age ranged from 1 month 17 days to 14 years, with a median age of 4 years (Table 1). There were 17 infants, 45 children in early childhood, 23 preschool children and 24 school-age children. All patients were full term, with a normal birth history and past medical history.

**Table 1:** General characteristics and clinical characteristics of the 109 cases

General characteristics	Total, *n* = 109
Male sex, n (%)	64 (58.7)
Female, n (%)	45 (41.3)
**Onset age, median (range)**	4 years (1 month 17 days to 14 years)
Infant, n (%)	17 (15.6)
1–3 years, n (%)	45 (41.3)
4–6 years, n (%)	23 (21.1)
7–15 years, n (%)	24 (22)
Follow-up, median (range)	6.5 years (1 to 15 years)
**Sub-type, n (%)**	
Caudate head	38 (34.9)
Thalamus	21 (19.2)
Lenticular nucleus	50 (45.9)
**Symptom, n (%)**	
Hemiplegia	107 (98.2)
Facioplegia	46 (42.2)
Aphasia	27 (24.8)
Seizures	18 (16.5)
Headache/vomiting	9 (8.3)
**Aetiology, n (%)**	
Mild trauma	53 (48.6)
Bilateral basal ganglia calcification	21 (19.3)
Increased homocysteine	8 (7.3)
Vasculitis	4 (3.7)
Infection	4 (3.7)
Congenital vascular malformation	5 (4.6)
Cardiac disease	5 (4.6)
Cardiac interventional surgery	2 (1.8)
Atrial myxoma	2 (1.8)
Tetralogy of Fallot	1 (0.9)
Unknown	17 (15.6)

Ninety-nine patients had major complaints of unilateral body weakness, 6 had focal seizures, 3 had facial paralysis, 3 had headache and vomiting, and 1 had aphasia.

The time from onset to diagnosis ranged from 1 h to 300 days, with a mean of 4 days. There were 13 cases in which the time from onset to diagnosis was less than 12 h and 29 cases in which the time period was less than 24 h. All cases underwent a professional physical examination. A total of 107 cases experienced different severities of weakness in the unilateral limbs when diagnosed, often reaching the peak level within 24 h after onset. All 107 patients had lower strength in their upper limbs compared to that in their legs (0-V level muscle strength). Two patients had headache, vomiting and focal seizures but no limb weakness. Forty-six patients had central facial paralysis. Twenty-seven patients had aphasia; these patients were able to understand words and presented speechlessness or slow speech speed. Nine patients exhibited headaches and vomiting. Eighteen patients had focal seizures.

A total of 109 cases were examined by CT, MRI, MRA, video electroencephalography (VEEG), echocardiography, electrocardiography, routine blood and biochemical examination, homocysteine evaluation, protein S evaluation, protein C evaluation, routine cerebral spinal fluid (CSF) evaluation, CSF biochemistry evaluation and virus antibody evaluation (Epstein–Barr virus[EBV]/ herpes simplex virus[HSV]/measles).

**Fig. 1:**
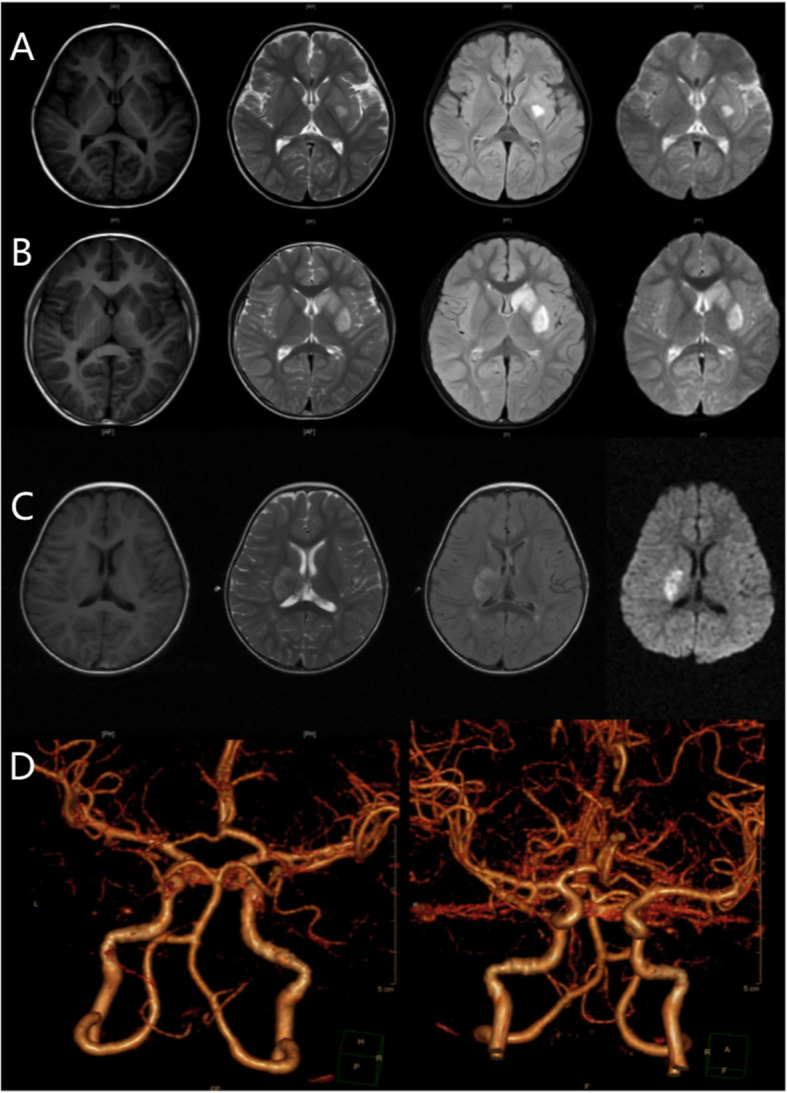
**a** Case 1, a 2-year-old boy presented with right facial paralysis accompanied by right limb fatigue for 48 h. The muscle strength of his right upper and lower limbs was grade II. The strength of his left limbs was normal. The right nasolabial groove was shallow, and the angle of his mouth was skewed to the left. MRI results showed that the left lenticular nucleus was involved. **b** Case 2, an 8-year-old boy had sudden hemiplegia of his right limb for 6 h without any inducement. His pronunciation was slurred. The muscle strength of his right upper and lower limbs was grade 0. The strength of his left limbs was normal. His right nasolabial groove was shallow. MRI results showed that the left lenticular nucleus with the caudate head was involved. **c** Case 3, a 1-year-old girl had left limb weakness for 48 h after falling from a height of 50 cm. The muscle strength of her left upper and lower limbs was grade III. Her left nasolabial groove became shallow, and the angle of her mouth was skewed to the right. The right lenticular nucleus with thalamus involvement was shown on MRI. The images from left to right were acquired by T1, T2, FLAIR and DWI. **d** The MRA of case 2 showed that the left anterior A1 segment was slightly thinner than the right anterior A1 segment, and the left lenticular artery was not clear

MRI of the brain could be normal within 6 h, with a low T1 signal and a high T2 signal in fluid-attenuated inversion recovery (FLAIR) and diffusion-weighted imaging (DWI), and the high FLAIR and DWI signals could fade out with time. All 109 cases had no lesions in brain regions other than the basal ganglia and thalamus. All 109 cases had lentiform nucleus lesions: 50 cases only had lesions in the lenticular nucleus (lenticular nucleus group), as shown in Fig. 1a; 21 cases had lesions in both the lenticular nucleus and caudate head (caudate head group), as shown in Fig. 1b; and 38 cases had lesions in both the lenticular nucleus and thalamus (thalamus group), as shown in Fig. 1c. MRA results were unremarkable, except for some occasional mild vascular malformations; for example, some lesions were slightly thin on one side of the anterior cerebral artery, as shown in Fig. 1d (Fig. 1).

CT of the brain identified bilateral punctate calcification of the basal ganglia in 21 cases.

VEEG results showed bilateral voltage asymmetry, such as the affected side was associated with a lower voltage, or the advantage of the occipital region was not obvious. Children with focal seizures exhibited discharge in the EEG. Through routine echocardiography detection, we found 2 cases of congenital heart disease after intervention, 1 case of tetralogy of Fallot, and 2 cases of atrial myxoma. The acute phase coincided with increased homocysteine in 8 cases, and the level of homocysteine was 50 μmol/l in one 8-year-old patient. This patient had a second basal ganglia ischaemic stroke 1 year later, and at the acute phase of the second time, the level of homocysteine was 22 μmol/l. We administered folate 2 mg qd, vitamin B6 10 mg qd and vitamin B12 5 mg qd; later, the patient’s homocysteine level was normal, and subsequent stroke did not occur. The other 7 patients had a mild level of hyperhomocysteinemia during the acute phase, ranging from 15.58 to 22.5 μmol/l (mean ± SD, 18.09 ± 2.6); upon examination 1 week later, all patients exhibited normal levels. All the cases had a blood and urine metabolism screening with normal results. All children had blood pressure in the normal range for their age.

### Etiology

A total of 53/109 children (48.6%) had a definite history of trauma within 12 h before the onset of the disease, but the injuries were always very mild, such as that acquired by falling from the bed. A total of 21/109 children (18.3%) had bilateral basal ganglia calcification; blood and urine metabolic screening and homocysteine tests were conducted to exclude genetic metabolic diseases. Five patients had congenital vascular malformations. Eight/109 (7.3%) patients had increased homocysteine in the acute phase, and levels returned to normal 1 week later. A total of 4/109 children (3.7%) had varicella infection 2 weeks before the disease, and 4/109 children (3.7%) had allergic purpura before the disease. Also, 2/109 children (1.8%) had an interventional therapy for congenital heart disease before stroke, 1/109 children (0.9%) had tetralogy of Fallot, 2/109 children (1.8%) had atrial myxoma, and 19/109 children (17.4%) cases had unknown aetiology. There were 10/109 (9.2%) patients with more than one stroke: 4/38 (10.5%) were in the thalamus group, 4/50 (8%) were in the lenticular nucleus group, and 2/21 (9.5%) were in the caudate head group. There was no significant difference in the incidence rate of each group.

### Treatment

Twenty-seven patients received aspirin antiplatelet therapy, 25 patients received low molecular weight heparin calcium anticoagulant therapy, 37 patients received antiplatelet and anticoagulant therapy, and 20 patients received only symptomatic and supportive therapy.

### Follow-up

None of the 109 cases were lost to follow-up, and the follow-up rate was 100%. The follow-up time was 1–15 years, with a median time of 6.5 years.

#### Limb paralysis

The patients with the fastest recovery of muscle strength recovered to grade 5 within 1 day, while those with slow recovery reached grade 5 within 1 month after the onset of disease.

There was no significant difference in the time required for muscle strength to recover to grade 5.

#### Fine motor dysfunction

Most of the children exhibited different degrees of fine motor disorder and dystonia on the affected side limbs. As time passed, the children’s fine motor disorder and dystonia symptoms gradually eased, and the time required to recover their fine motor skills was approximately 2–3 years or longer. Fine motor disorders and dystonia may exist for a long time. By the end of the follow-up period, all the children had no obvious dystonia, but 5/109 (4.6%) children had changed their handedness, manifesting as a child with a right-hand advantage changing to having a left-hand advantage. Regarding children who changed their handedness, a total of 3/21 (14.2%) patients were in the caudate head group, 1/50 (2.0%) were in the lenticular nucleus group, and 1/38 (2.6%) were in the thalamus group.

#### Facial paralysis

Most of the children with basal ganglia ischaemic stroke recovered completely from facial paralysis, with the shortest recovery time being 1 week and the longest recovery time being 3 months. There was no significant difference in the recovery time regarding facial paralysis among the groups.

#### Convulsion and aphasia

Children recovered quickly from focal convulsion, and those with abnormal EEG results were controlled within 1 week after treatment with oxcarbazepine or levetiracetam. Patients without abnormal EEG results recovered within 1 week without targeted treatment. Similarly, children recovered from aphasia within 2 weeks to 1 month without special treatment.

#### Overall prognosis

To evaluate the overall prognosis, the mRS score was calculated at the end of follow-up. The results showed that the scores of all 109 cases were less than or equal to 2 points, with an average of 0.62 points. The mean score was 0.63 points in the thalamus group, 0.65 points in the caudate head group, and 0.62 points in the lenticular nucleus group. The details are shown in Table 2. Statistical analysis of the relationship between the scores and the affected sites or treatment methods showed no statistical correlation.

**Table 2:** The mRS score of each group, n (%)

	0	1	2	Mean score
Caudate head	7 (35)	13 (65)	0 (0)	0.65
Lenticular nucleus	21 (41)	27 (54)	2 (4)	0.62
Thalamus	15 (39)	22 (58)	1 (3)	0.63
Total	43 (39)	62 (57)	3 (3)	0.62

*One-way ANOVA, *p* = 0.9782

#### Anxiety

To evaluate the anxiety of children in each group, the SCAS-P was administered to parents with children older than 6 years at the end of follow-up in each group. There were 15 children over 6 years old in the caudate head group, 43 in the lenticular nucleus group and 28 in the thalamus group. Sex, age, parents’ age, parents’ education and parents’ work conditions affected the children’s SCAS-P score. To exclude the influence of the above factors, we performed statistical analysis, and no significant differences among groups in our sample were found in sex, age, parents’ age, parents’ education or parents’ work conditions among the groups (Table 3, ordinary one-way ANOVA and Bartlett’ s test, *p* > 0.05).

**Table 3:** Demographic characteristics of children over 6 years old during follow-up

Demographic characteristics			
Characteristic	Caudate head	Lenticular nucleus	Thalamus
n	15	43	28
Male, n (%)	9 (60.0)	26 (60.5)	18 (64.2)
Follow-up age, n (%)			
6–12 years	10 (66.7)	22 (51.2)	20 (71.4)
13–18 years	5 (33.3)	19 (44.2)	8 (28.6)
19 years	0	2 (4.6)	0
Parent’s age range (years)	32–57	30–61	29–58
Parent’s education, n (%)			
Elementary school or less	1 (6.70)	1 (2.30)	1 (3.60)
Junior high school	4 (26.70)	10 (23.30)	7 (25.00)
High school	5 (33.30)	15 (34.90)	9 (32.10)
College/university or above	5 (33.30)	17 (39.50)	11 (39.30)
Occupation, n (%)			
Unemployed	2 (13.30)	7 (16.30)	4 (14.30)
Working-class jobs	6 (40.00)	18 (41.80)	11 (39.30)
Professional, managerial, or technical position	7 (46.60)	18 (41.80)	13 (46.40)

**Fig. 2:**
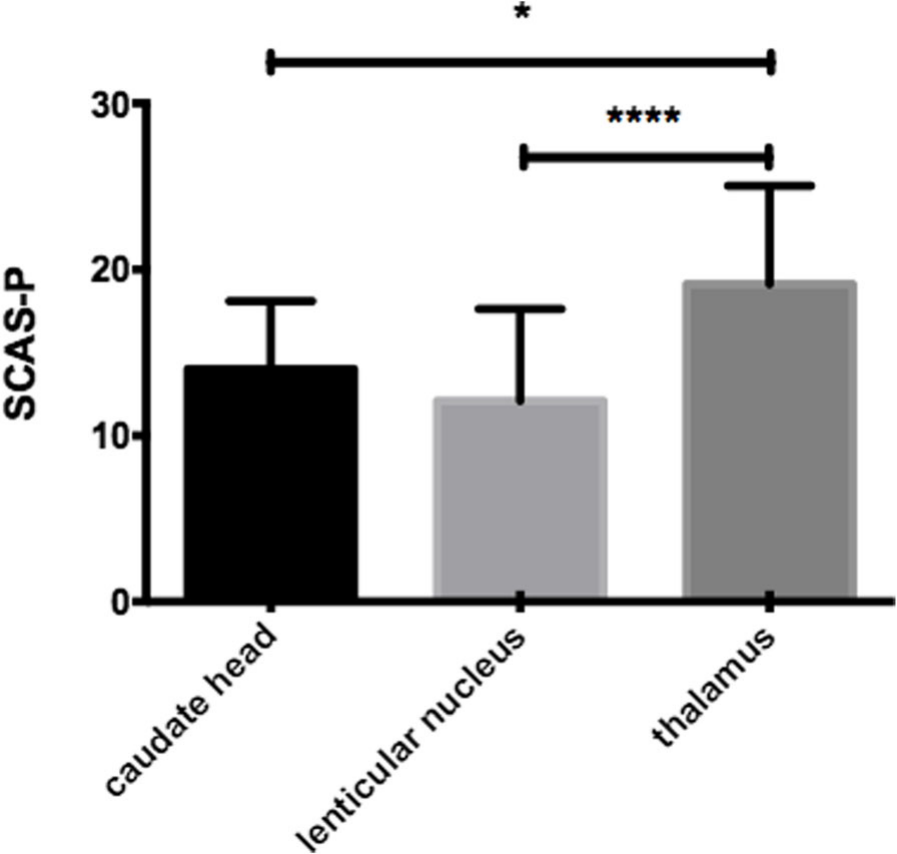
The SCAS-P score of each group. The SCAS-P score of the thalamus group was higher than that of the lenticular nucleus group and caudate head group, and the difference was statistically significant* *p* < 0.05, **** *p* < 0.0001

The SCAS-P scores were 14.00 ± 1.181 (mean ± SE, *n* = 12) in the caudate head group, 12.08 ± 0.877 (*n* = 43) in the lenticular nucleus group, and 19.12 ± 1.185 (*n* = 25) in the thalamus group. The SCAS-P score of the thalamus group was higher than that of the lenticular nucleus group and caudate head group, and the difference was statistically significant (as shown in Fig. 2).

## Discussion

This study found that children with basal ganglia ischaemic stroke accounted for 14.2% of children with ischaemic stroke during the same period. Basal ganglia ischaemic stroke was more prevalent in early children (1–3 years old) (41.3%), and basal ganglia ischaemic stroke was more common in male children than in female children (1.4:1). These results are consistent with previous studies [12].

Limb weakness was the main reason for treatment in children with basal ganglia ischaemic stroke, followed by facial paralysis and focal seizures. Because limb weakness was not obvious in infants and early children, it was not easy to detect in the early stage of the disease; therefore, the time from onset to treatment was longer. The average time for treatment in this group of patients was 4 days, which was longer than the optimal time window when considering thrombolysis.

All 107 patients with limb weakness had lower power in their upper limbs than that in their legs (0–5 level muscle strength). This difference in limb strength occurs because there are more cortical motor projection nerve fibres in the upper limbs than in the lower limbs through the basal ganglia. Therefore, it is necessary to consider diseases other than basal ganglia ischaemic stroke if the child’s upper limb muscle strength is greater than his or her leg strength.

In terms of aetiology, trauma and bilateral basal ganglia calcification are the most common causes. Vascular malformation, precursor infection, vasculitis and heart disease are other causes of basal ganglia stroke in children. In this study, 53/109 (48.6%) of the children with basal ganglia stroke had a trauma within 12 h before the onset of the disease, indicating that trauma is the cause of ischaemic stroke in the basal ganglia. The cause of ischaemic stroke in the basal ganglia may be related to the anatomical characteristics of the lenticular artery (also known as the lateral central artery). Vincentelli et al. found that the lenticular artery did not directly enter the brain tissue after it exited the middle cerebral artery but went through a section in the lateral fissure—the extracerebral segment—and then turned upward to supply blood to the basal ganglia and internal capsule. In children, the extracerebral segment of the lenticular artery is short and straight, its exit angle is nearly a right angle, and its tension is high. In adults, the external segment of the lenticular artery is long and curved, with an acute angle, and relatively loose. Therefore, the extracerebral segment of the lenticular artery in children is more vulnerable to torsion or shear stress. In addition, the development of sphenoid bone in children is now complete; it cannot completely cover the temporal lobe, which makes the subarachnoid space relatively wide, consequently making the brain tissue and skull base more likely to undergo large horizontal displacement upon an acceleration or deceleration injury so that the lenticular artery is damaged [9]. A study reported that a child had an abnormal lenticular artery at the age of 1 month, and several months later, an ischaemic stroke was observed after mild trauma; at the same time, lenticulostriate artery mineralization was demonstrated [13]. Ivanov et al. reported trauma-associated ischaemic stroke in an 8-month-old infant with pre-existing lenticulostriate vasculopathy [4]. This indicates that the underlying lenticulostriate vasculopathy predisposed the infant to, or worsened, vascular obstruction caused by head trauma. It has been suggested that the abnormal structure of the lenticulostriate artery could promote the occurrence of basal ganglia ischaemic stroke [14]. The relationship between basal ganglia calcification and ischaemic stroke is still controversial. Some hold the opinion that basal ganglia calcification may be asymptomatic and idiopathic basal ganglia calcification; this inference was determined because even if a normal person carries undergoes CT examination, a certain proportion of bilateral basal ganglia calcification is evident [15].

However, some studies have found that basal ganglia calcification is actually the manifestation of mineralizing angiopathy of lenticulostriate arteries [4]. In this study, 21/109 (18.3%) children had bilateral basal ganglia calcification, and the incidence was almost consistent with previous studies [13, 16]. All patients underwent blood and urine metabolic screening to ensure that basal ganglia calcification caused by genetic metabolic diseases was excluded. The pathophysiology of this phenomenon is not very evident at this point and might require further research. In this study, routine biochemical and viral antibody detection of cerebrospinal fluid was normal in all children, suggesting that, unlike other regions of children with ischaemic stroke, infection is not the main cause of basal ganglia stroke in children [12]. Eight patients had increased homocysteine levels in the acute phase, and a week later, all underwent blood and urine metabolic screening to eliminate the possibility of genetic metabolic diseases. Homocysteine can increase the incidence of stroke in many ways. It can increase the proliferation of smooth muscle cells and increase the production of collagen. The free radicals formed in the process of oxidation can cause oxidative stress that damages the endothelium. Obvious platelet aggregation may be secondary to the direct promoting effect of homocysteine or the inhibition of platelets mediated by the endothelium [17, 18]. Some studies have found that folic acid, vitamin B6 and vitamin B12 can reduce the level of homocysteine, thus reducing small vessel stroke, which is consistent with the findings of the children in this study [19]. Therefore, for children with basal ganglia ischaemic stroke, the level of homocysteine should be routinely screened and followed up. If necessary, B vitamins could be given to reduce homocysteine.

In addition, there were no cases of sickle cell anaemia in the 109 children in the present study. The aetiology of ischaemic basal ganglia stroke in Chinese children is different from that in children in European and American countries, which may be related to differences among ethnic groups [3]. The incidence of sickle cell anaemia in the Asian population is relatively low. None of the patients were diagnosed with cytomegalovirus infection. IgM and PCR results were negative. This is not consistent with previous studies that showed a high incidence of cytomegalovirus infection in children with basal ganglia stroke. Of course, this discrepancy may be due to the difference in age among the participants in previous studies and in the group of children in the present study [14].

There is no consensus on whether thrombolytic therapy should be carried out as soon as possible in children with basal ganglia ischaemic stroke. In our study, although most children missed the opportune window for thrombolytic therapy, it is gratifying that though all of our children did not receive thrombolytic therapy, the mRS scores were less than or equal to 2 points; it was also notable that there was no significant difference in the mRS scores of children with single antiplatelet, single anticoagulant, combined antiplatelet anticoagulation or only support treatment, which is consistent with previous studies [14]. Of course, the recommendation for treatment needs to be supported by further large-scale randomized controlled trials.

Children with basal ganglia ischaemic stroke recovered their muscle strength rapidly, and the muscle strength of their affected limbs recovered to grade V within 1 month. Prashant Jauhari el al. hypothesized that transient vasospasm secondary to trauma-induced stretching of lenticulostriate vessels may have led to a more favourable outcome in these children [20].

Fine motor problems may remain in children with basal ganglia ischaemic stroke. In this group of cases, fine motor problems and dystonia were not obvious at the follow-up 1 year after stroke in any of the groups based on lesion location, but a few children experienced changes in their fine motor skills that led to changes in handedness. The caudate head group had a higher incidence of a change in handedness, which may be related to the fact that the head of the caudate nucleus governs autonomous movement. It has been found that damage to the caudate nucleus of the cat leads to an abnormal posture of the contralateral limbs, abnormal precision and abnormal fine motion velocity [21]. Facial paralysis, convulsion and aphasia in children with basal ganglia ischaemic stroke was noted only in the acute stage. The above symptoms in this study were ameliorated within the first 3 months.

This study found that children with basal ganglia ischaemic stroke can lead to anxiety disorder, which is consistent with recent studies [8]. More interestingly, we found that children in the thalamus group had more severe anxiety.

The thalamus is a hub for all sensory information entering the brain.

Some functional nuclei of the thalamus, such as the paraventricular nucleus, regulate emotional responses to sensory stimuli [22, 23]. Because the inadequate emotional response to certain sensory stimuli is an important feature of post-traumatic stress syndrome (PTSD), the thalamus may play a role in the pathophysiology of PTSD. Indeed, recent studies have shown that thalamic neural circuits are involved in the response to threatening sensory stimuli and in fear extinction in PTSD [23, 24].

A large amount of evidence has shown that the thalamus plays a key role in regulating the function of the amygdala. The amygdala is closely related to the generation and regulation of anxiety emotion [22, 25]. Penzo MA et al. found that the thalamus regulates fear processing in the lateral division of the central amygdala in mice, thus coordinating conditioned fear [23]. Merijn Joling et al. found that the severity of anxiety symptoms showed a significant negative association with ^123^I-FP-CIT binding ratios in the thalamus in Parkinson’s disease patients, indicating that the integrity of both dopaminergic and serotonergic neurons in the thalamus is closely related to anxiety [26]. Min Lu et al. examined whole brain white matter alterations in young healthy individuals with high anxiety but without a history of neurological or psychiatric disorders via DTI (Diffusion Tensor Imaging) technology [27]. Individuals with high anxiety have white matter alterations in the thalamocortical circuit, and the altered white matter may be a vulnerability marker in individuals at high risk of clinical anxiety [28]. Mild stroke with basal ganglia region infarcts may be related to widespread abnormalities in white matter integrity [29].

Recent studies found that basal ganglia ischaemic stroke can lead to dystonia and that dystonia is associated with anxiety [7]. However, we found that dystonia was not obvious in almost all 109 children as of the follow-up time, indicating that dystonia is not the direct cause of anxiety in our study. Of course, the previous study included patients with cortical and basal ganglia involvement, which may lead to different results [7].

For the lacking of the control group in terms of cases without basal ganglia stroke or cases without stroke, our findings are applicable within basal-ganglia stroke population only.

## Conclusion

This study found that the overall prognosis of children with basal ganglia ischaemic stroke is good, but it may cause fine motor disorders and anxiety. When the caudate head affected, it is easy for the patient to develop fine motor disorders; furthermore, patients in which the thalamus is affected are prone to anxiety. For children with basal ganglia ischaemic stroke, in addition to limb rehabilitation and motor function evaluation, emotional fluctuation should also be closely monitored.

## Data Availability

Not applicable.

## Abbreviations

mRS: Modified Rankins score
EBV: Epstein-Barr virus
HSV: Herpes simplex virus
DTI: Diffusion tensor imaging
CT: Computerized tomography
MRI: Magnetic resonance imaging
MRA: Magnetic resonance angiography
FLAIR: Fluid-attenuated inversion recovery
DWI: Diffusion-weighted imaging
VEEG: Video electroencephalography
CSF: Cerebral spinal fluid
SCAS-P: The Spence Children’s Anxiety Scale for Parents
PTSD: Post-traumatic stress syndrome
